# Transversus Abdominis Plane Block With Liposomal Bupivacaine vs. Regular Anesthetics for Pain Control After Surgery: A Systematic Review and Meta-Analysis

**DOI:** 10.3389/fsurg.2020.596653

**Published:** 2020-11-05

**Authors:** Yi Zhu, Ting Xiao, Shuangquan Qu, Zheng Chen, Zhen Du, Jiangping Wang

**Affiliations:** Department of Anesthesiology, Hunan Children's Hospital, Changsha, China

**Keywords:** extended analgesia, local anesthetic, pain, opioids, surgery

## Abstract

**Background:** Transverse abdominal plane (TAP) blocks are used to provide pain relief after abdominopelvic surgeries. The role of liposomal bupivacaine (LB) for TAP blocks is unclear. Therefore, this study aimed to synthesize evidence on the efficacy of LB vs. regular anesthetics in improving outcomes of TAP block.

**Methods:** PubMed, Science Direct, Embase, Springer, and CENTRAL databases were searched up to July 24, 2020. Studies comparing LB with any regular anesthetic for TAP block for any surgical procedure and reporting total analgesic consumption (TAC) or pain scores were included.

**Results:** Seven studies including five randomized controlled trials (RCTs) were reviewed. LB was compared with regular bupivacaine (RB) in all studies. A descriptive analysis was conducted for TAC due to heterogeneity in data presentation. There were variations in the outcomes of studies reporting TAC. Meta-analysis of pain scores indicated statistically significant reduction of pain with the use of LB at 12 h (MD: −0.89 95% CI: −1.44, −0.34 I^2^ = 0% *p* = 0.01), 24 h (MD: −0.64 95% CI: −1.21, −0.06 I^2^ = 0% *p* = 0.03), 48 h (MD: −0.40 95% CI: −0.77, 0.04 I^2^ = 0% *p* = 0.03) but not at 72 h (MD: −0.37 95% CI: −1.31, 0.56 I^2^ = 57% *p* = 0.43). Pooled analysis indicated no difference in the duration of hospital stay between LB and RB (MD: −0.18 95% CI: −0.49, 0.14 I^2^ = 61% *p* = 0.27). LB significantly reduced the number of days to first ambulation postsurgery (MD: −0.28 95% CI: −0.50, −0.06 I^2^ = 0% *p* = 0.01).

**Conclusions:** Current evidence on the role of LB for providing prolonged analgesia with TAP blocks is unclear. Conflicting results have been reported for TAC. LB may result in a small reduction in pain scores up to 48 h but not at 72 h. Further, high-quality homogenous RCTs are needed to establish high-quality evidence.

## Introduction

Postsurgical pain can significantly impact patient satisfaction and overall quality of life (1). Literature suggests that optimal management of acute pain may also influence the development of chronic pain after the surgical procedure (2). Opioids are one of the most predominant drugs used for pain relief worldwide. However, side effects like nausea, vomiting, constipation, respiratory depression, etc. are commonly associated with their use. These adverse effects can increase patient morbidity while also adding to the overall healthcare costs (3). A multimodal approach for pain control is therefore recommended by balancing the benefits and adverse events of every drug or intervention.

The transverse abdominal plane (TAP) block was first described by Rafi et al. (4) in 2001 for pain relief after abdominopelvic surgeries. The technique involves infiltration of local anesthetic into the plane between the internal oblique and transversus abdominus muscles thereby blocking the neural afferents of the thoracoabdominal nerves originating from T6 to L1 spinal roots (5). Since then, TAP blocks have been used to provide postoperative pain relief after several surgical procedures like colorectal surgery, hernia repairs, prostatectomy, hysterectomy, cesarean sections, and sleeve gastrectomy (6–8). Cai et al. (6), in a recent systematic review and meta-analysis of 15 studies, have demonstrated that TAP provides more effective and steady analgesia compared to infiltration with local anesthetics in adult patients in the early postoperative period.

Regular anesthetics like bupivacaine are commonly used for TAP blocks to provide prolonged analgesia after surgery (9). Since regular bupivacaine (RB) provides pain relief for only up to 10 h after surgery, it is not surprising for trials to report reduced opioid consumption and lower pain scores only on the first postoperative day with the use of RB (10, 11). The use of infusion pumps or catheters may provide longer analgesia but are associated with patient discomfort and other complications (6). Liposomal bupivacaine (LB) is a multivesicular liposomal formulation of 1.3% bupivacaine capable of providing pain relief for up to 72 h (9). Several studies have assessed their efficacy for joint arthroplasties and other soft tissue surgeries (12). However, evidence of its prolonged efficacy has not been coherent. While some meta-analysis studies comparing LB vs. RB for pain relief after hip arthroplasty have indicated improved outcomes with LB (13), others have reported no difference in outcomes compared to regular anesthetics after shoulder surgeries (14). LB has been used by several clinicians for administering the TAP block. However, to the best of our knowledge, no review has attempted to synthesize evidence on its efficacy vs. regular anesthetics. Therefore, this review aimed to conduct a systematic literature search and pool data to answer the following clinical question: Does the use of LB vs. regular anesthetics for the TAP block improve clinical outcomes in adults undergoing abdominopelvic surgeries?

## Materials and Methods

### Inclusion Criteria

We framed our inclusion criteria based on the Population, Intervention, Comparison, Outcome, and Study design (PICOS) outline. Studies conducted on patients undergoing any type of surgical procedure and receiving transverse abdominal pain (TAP) blocks for pain control were to be included (*Population*). The study *intervention* was to be the use of LB *compared* to any regular anesthetic. *Outcomes* measured were to be total analgesic consumption or pain scores. We included only randomized controlled trials (RCTs), prospective nonrandomized, and retrospective single-center case-control studies. The following studies were excluded from the review: (1) studies not reporting outcomes, relevant outcomes, or not mentioning the distribution of data using standard deviations (SD), standard errors (SE), range or 95% confidence intervals (CI); (2) studies including <10 patients per group; (3) studies utilizing continuous anesthetic infiltration using catheters; (4) studies using other anesthesia regimens in the control group like epidural anesthesia; (5) interrupted time series design studies and studies comparing outcomes among different healthcare setups; and (6) single-arm studies, case series, case reports, review articles were also excluded.

### Search Strategy

Two reviewers independently conducted electronic searches of PubMed, ScienceDirect, Embase, Springer, and CENTRAL databases from inception up to July 24, 2020 using the following search terms: “transverse abdominal block,” “transverse abdominal plane,” “bupivacaine,” “liposomal bupivacaine,” “anesthesia,” “analgesia,” and “block.” The literature search was restricted to English language publications. The search strategy and results of the PubMed database are presented in Supplementary Table S1. After evaluating the studies at the title and abstract level, full texts of selected articles were scanned for inclusion in the review. References of included studies were hand searched for identification of any missed out studies. Any disagreements were resolved by discussion between the two reviewers. Guidelines of the PRISMA statement (Preferred Reporting Items for Systematic Reviews and Meta-analyses) (15) and Cochrane Handbook for Systematic Reviews of Intervention (16) were followed during the conduct of this review.

### Data Extraction and Outcomes

Data were extracted from the included studies by two reviewers independently. The following details were obtained using a pre-prepared data collection form: authors, publication year, study location, surgery type, sample size, mean age, intervention and control protocol, use of other analgesics, and study outcomes. The primary outcome of interest of this review was the total analgesic consumption and pain scores. The secondary outcomes were the length of hospital stay and time for ambulation postsurgery.

### Risk of Bias

All included RCTs were assessed for bias using the Cochrane Collaboration risk assessment tool (17). Studies were rated as low risk, high risk, or unclear risk of bias for each of the following variables: random sequence generation, allocation concealment, blinding of participants and personnel, blinding of outcome assessment, incomplete outcome data, and selective reporting. For non-RCTs, the risk of a bias assessment tool for nonrandomized studies (RoBANS) was used (18). Studies were assessed for the selection of participants, confounding variables, intervention measurements, blinding of outcome assessment, incomplete outcome data, and selective outcome reporting.

### Statistical Analysis

As all outcomes of the review were continuous variables, they were summarized using the mean difference (MD) with 95% confidence intervals (CI), if the data was measured on the same scale. In case different scales were used, standardized mean difference (SMD) was to be calculated with 95% CI. We used a random-effects model to calculate the pooled effect size for all our analyses. Heterogeneity was assessed using the I^2^ statistic. I^2^ values of 25–50% represented low values of 50–75% medium, and more than 75% represented substantial heterogeneity. For studies not reporting continuous variables as mean and standard deviation scores, they were calculated using methods reported by Wan et al. (19). We used the software Engauge Digitizer to extract numerical data if outcomes were reported only graphically. Due to the inclusion of fewer than 10 studies in the review, funnel plots were not used to assess publication bias. We used the software “Review Manager” (RevMan, version 5.3; Nordic Cochrane Centre [Cochrane Collaboration], Copenhagen, Denmark; 2014) for the meta-analysis.

## Results

**Figure 1:**
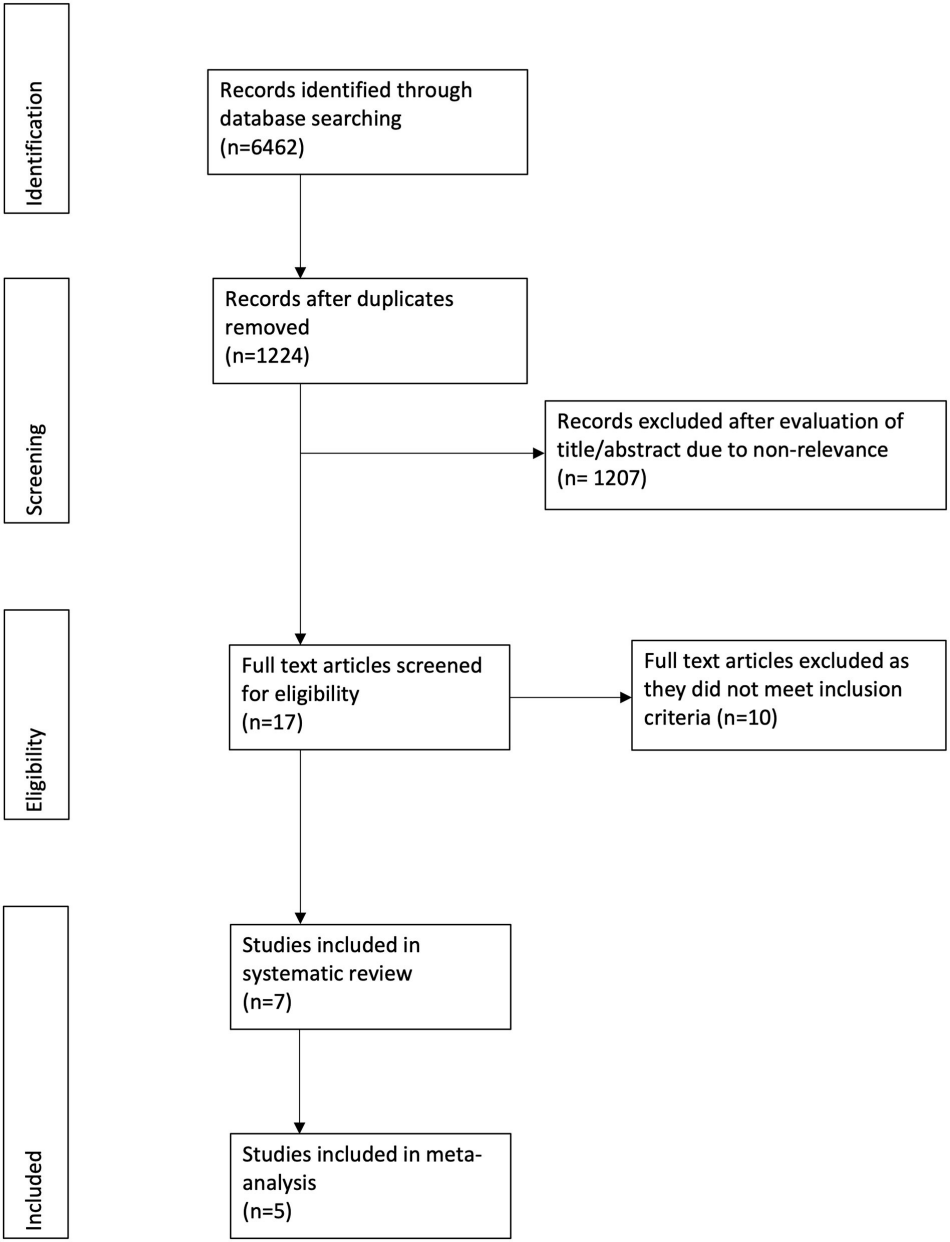
Study flow chart.

Figure 1 depicts the study flow chart. Seventeen studies were assessed by their full texts. Ten were excluded as per the reasons mentioned in Table 1 (20–29). A total of seven studies were included in this systematic review and meta-analysis (30–36). Details of the included studies are presented in Table 2. All studies were conducted in the United States. Five studies were RCTs (30–34), one was a prospective (35), while another was a retrospective study of a prospectively collected database (36). Ultrasound (US) guidance was used for the TAP blocks in four studies (30, 33, 34, 36). The amount of LB and RB varied across studies along with variations in the analgesic regimen.

**Table 1:** List of excluded studies.

**References**	**Reason for exclusion**
Jablonka et al. (20)	Continuous infusion of RB with catheter in control group
Fields et al. (21)	Interrupted time series study with comparison between different hospital
Hutchins et al. (22)	Control group received only port-site infiltration of RB with no TAP block
Gatherwright et al. (23)	Less than 10 patients and not reporting SD/SE values of outcomes
Hutchins et al. (24)	Intrathecal morphine in control group
Yeap et al. (25)	Continuous infusion of RB with catheter in control group
Sternlicht et al. (26)	No control group receiving RB
Feierman et al. (27)	No control group receiving RB
Ayad et al. (28)	Control group receiving epidural anesthesia
Felling et al. (29)	Control group receiving epidural anesthesia

*RB, regular bupivacaine; SD, standard deviation; SE, standard error; TAP, transverse abdominal plane*.

**Table 2:** Characteristics of included studies.

**References**	**Study type**	**Surgery type**	**Sample size**		**Mean age**		**LB protocol**	**RB protocol**	**Analgesia regimen**
			**LB**	**RB**	**LB**	**RB**			
Nedeljkovic et al. (30)	RCT	Cesarean section	91	83	34^*^	33^*^	US-guided block with 266 mg of LB and 50 mg RB diluted in 60 ml of saline	US-guided block with 50 mg RB diluted in 60 ml of saline	Epidural anesthesia with 1.4–1.6 ml of hyperbaric 0.75% RB with 150 μg of morphine and 15 μg of fentanyl. Postoperatively acetaminophen and ibuprofen for up to 72 h or until hospital discharge
Wong et al. (31)	RCT	Roux-en-Y gastric bypass, Sleeve gastrectomy, or sleeve-to-bypass conversion	75	73	42.1 ± 9.8	39.4 ± 10.9	Laparoscopic block with 20 ml of LB, 0.25% bupivacaine, 100 ml saline	Laparoscopic block with 50 ml of 0.25% RB, 100 ml saline	Fentanyl PCA at 10 μg at a maximum of every 10 min × 24 h, acetaminophen with codeine elixir (360 mg/36 mg) oral × 4 h, ketorolac 30 mg IV × 6 h, and dilaudid 0.4 mg IV × 3 h or morphine 4 mg IV × 4 h as needed for breakthrough pain
Ha et al. (32)	RCT	Abdominally based autologous breast reconstruction	22	22	49 ± 9.2	49 ± 10	Under direct vision with 266 mg of LB diluted in 30 ml of saline	Under direct vision with 75 mg of RB diluted in 30 ml of saline	Preoperative paravertebral blocks with 15 ml of 0.5% bupivacaine. Acetaminophen, celecoxib, OxyContin before the blocks and postoperatively for preemptive analgesia. On day of surgery, 1 mg of hydromorphone available every hour for rescue analgesia. From day 1, 5–10 mg of oral oxycodone x 3 h, and 0.5 mg of hydromorphone IV × every hour as needed for breakthrough pain
Guerra et al. (35)	Prospective non-randomized	Laparoscopic colectomy	50	50	57.88 ± 1.56	58.37 ± 1.91	Laparoscopic block with 20 ml LB, 40 ml RB and 20 ml saline	Laparoscopic block with 60 ml RB and 20 ml saline	Scheduled oral acetaminophen, ibuprofen, and gabapentin. IV Dilaudid or morphine ordered for breakthrough pain.
Stokes et al. (36)	Retrospective	Colorectal surgery	303	104	53.8 ± NR	51.8 ± NR	US-guided block with 10 ml of LB with 5 ml of 0.25% RB on each side	US-guided block with 20 ml of 0.25% RB on each side	Opioids allowed postoperatively. Ketorolac or ibuprofen ordered as needed every 6 h up to 5 days
Hutchins et al. (33)	RCT	Donor nephrectomy	30	29	41 ± 12.5	38 ± 12.6	US-guided block with 10 ml of 1.3% LB with 20 ml saline on each side	US-guided block with 30 ml of 0.25% RB with 1:200,000 epinephrine	IV or oral opioids and ketorolac given when patients experienced moderate to severe postoperative pain. IV opioids used were fentanyl, hydromorphone or morphine, and oral opioids were hydromorphone, hydrocodone or oxycodone.
Hutchins et al. (34)	RCT	Robotic hysterectomy	28	30	60.5 ± 10.8	56.8 ± 10	US-guided block with 10 ml of 1.3% LB with 20 ml saline on each side	US-guided block with 30 ml of 0.25% RB with 1:200,000 epinephrine	Hydromorphone or fentanyl at the discretion of registered nurse anesthetist or anesthesiology resident

* *Median age*.

*RCT, randomized controlled trial; US, ultrasound; LB, liposomal bupivacaine; RB, regular bupivacaine; PCA, patient controlled anesthesia; IV, intravenous*.

### Primary Outcomes

The total analgesic consumption postsurgery was reported by all included studies. However, there was significant heterogeneity in the presentation of data (least-square means, median, and means) with the outcome being measured for different time intervals. Hence, only a descriptive analysis was carried out. Results reported by the included studies for total analgesic consumption are presented in Table 3. Except for the studies of Wong et al. (31) and Ha et al. (32), which did not report any statistically significant difference in total analgesic consumption between the two groups, all studies reported significantly lower consumption of analgesics in the LB group at some time interval.

**Table 3:** Outcome of “total analgesic consumption” reported by the included studies.

**References**	**Analgesic**	**Duration**	**Results**
Nedeljkovic et al. (30)	Morphine equivalents	0–24 h	No statistical significant difference between the two groups (*p* = 0.54)
		0–48 h	Significantly reduced analgesic consumption in LB group (*p* = 0.01)
		0–72 h	Significantly reduced analgesic consumption in LB group (*p* = 0.01)
		0–7 days	Significantly reduced analgesic consumption in LB group (*p* = 0.01)
		0–14 days	No statistical significant difference between the two groups (*p* = 0.54)
Wong et al. (31)	Fentanyl equivalents	0–48 h	No statistical significant difference between the two groups (*p* = 0.97)
	Total PCA	0–48 h	No statistical significant difference between the two groups (*p* = 0.98)
Ha et al. (32)	Morphine equivalents	PACU	No statistical significant difference between the two groups (*p* = 0.38)
		Total hospitalization time	No statistical significant difference between the two groups (*p* = 0.98)
Guerra et al. (35)	Morphine equivalents	Total hospitalization time	Significantly reduced analgesic consumption in LB group (*p* = 0.0002)
Stokes et al. (36)	Morphine equivalents	0–12 h	Significantly reduced analgesic consumption in LB group (*p* < 0.05)
		0–24 h	Significantly reduced analgesic consumption in LB group (*p* < 0.05)
		0–36 h	Significantly reduced analgesic consumption in LB group (*p* < 0.05)
		0–48 h	No statistical significant difference between the two groups (*p* = NR)
		0–60 h	No statistical significant difference between the two groups (*p* = NR)
Hutchins et al. (33)	Fentanyl equivalents	0–24 h	No statistical significant difference between the two groups (*p* = NR)
		24–48 h	No statistical significant difference between the two groups (*p* = NR)
		48–72 h	Significantly reduced analgesic consumption in LB group (*p* = 0.03)
Hutchins et al. (34)	Morphine equivalents	0–24 h	Significantly reduced analgesic consumption in LB group (*p* = 0.02)
		24–48 h	Significantly reduced analgesic consumption in LB group (*p* = 0.01)
		48–72 h	No statistical significant difference between the two groups (*p* = 0.29)
		0–72 h	Significantly reduced analgesic consumption in LB group (*p* = 0.002)

*PACU, postoperative acute care unit; PCA, patient-controlled analgesia; NR, not reported*.

**Figure 2:**
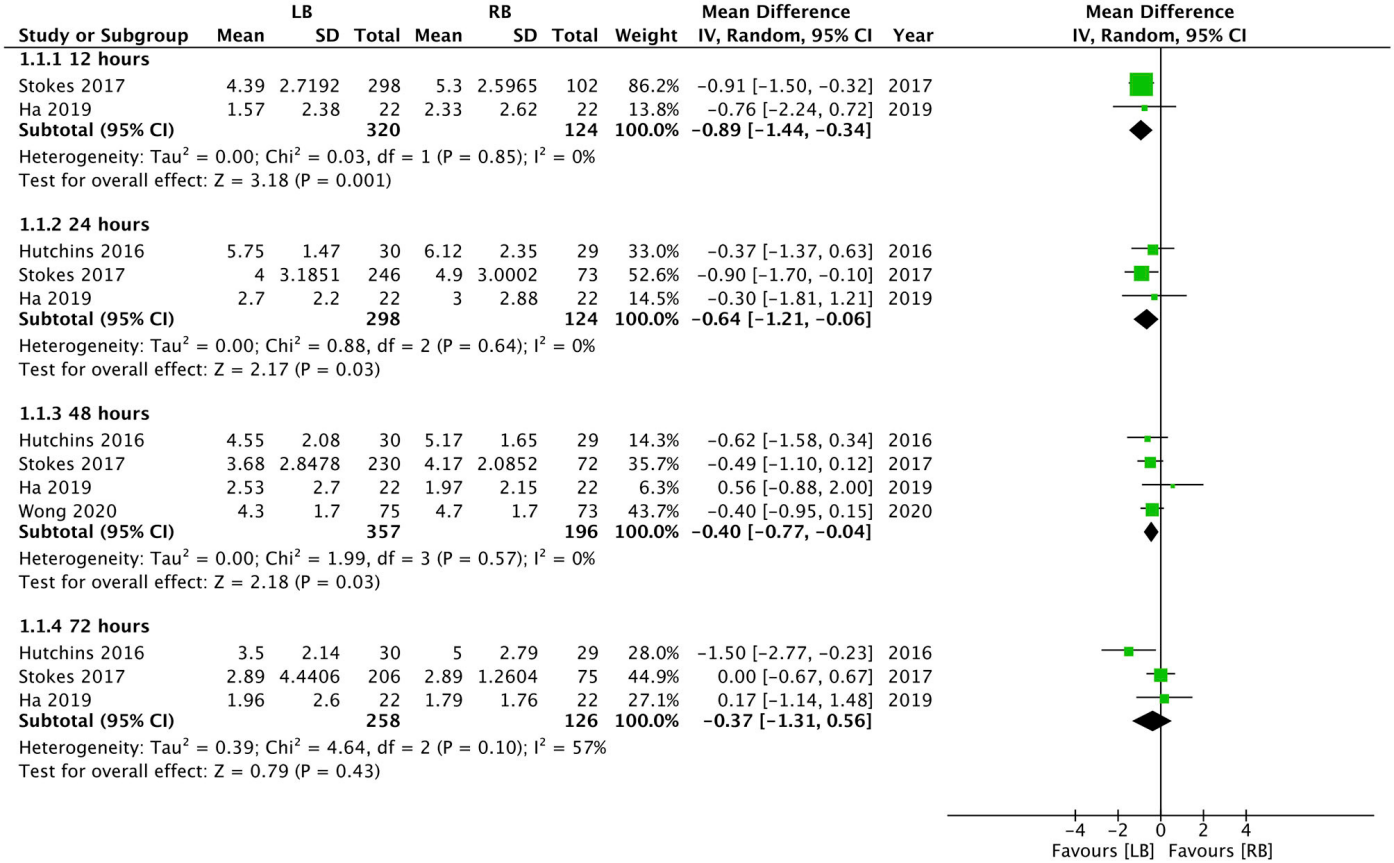
Forest plot of pain scores for liposomal bupivacaine (LB) vs. regular bupivacaine (RB) for transverse abdominal plane (TAP) block at different time intervals.

Except for the study of Guerra et al. (35), all studies included pain scores as one of their outcomes. Hutchins et al. (34) did not report the numerical or graphical data on pain scores in their article, while Nedeljkovic et al. (30) did not report data as mean and SD. Data from the remaining four studies were pooled for a meta-analysis. Our results indicated statistically significant reduction of pain with the use of LB for TAP block at 12 h (MD: −0.89, 95% CI: −1.44, −0.34 I^2^ = 0% *p* = 0.01), 24 h (MD: −0.64, 95% CI: −1.21, −0.06 I^2^ = 0% *p* = 0.03), 48 h (MD: −0.40, 95% CI: −0.77, −0.04 I^2^ = 0% *p* = 0.03) but not at 72 h (MD: −0.37, 95% CI: −1.31, 0.56 I^2^ = 57% *p* = 0.43) (Figure 2). Nedeljkovic et al. (30) in their study reported a statistically significant difference in pain scores between LB and RB at all time intervals up to 72 h. Hutchins et al. (34) reported significantly lower maximal pain scores in the LB group at 24-, 48-, and 72-h time points compared to the RB group.

### Secondary Outcomes

**Figure 3:**
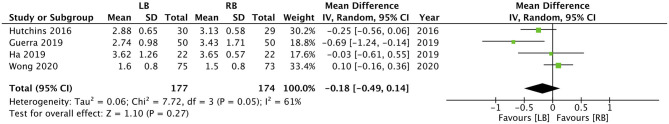
Forest plot of length of hospital stay in days for LB vs. RB for TAP block.

**Figure 4:**
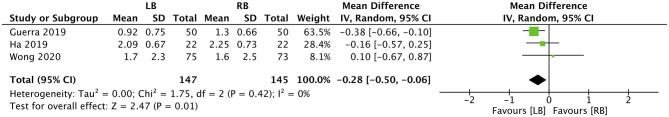
Forest plot of time to unassisted ambulation in days for LB vs. RB for TAP block.

Four studies reported data on the length of hospital stay. Our pooled analysis indicated that the use of LB does not reduce the duration of hospital stay in days (MD: −0.18, 95% CI: −0.49, 0.14 I^2^ = 61% *p* = 0.27) (Figure 3). However, when data from three studies were analyzed, our results indicated that LB may significantly reduce the number of days to first ambulation postsurgery (MD: −0.28, 95% CI: −0.50, −0.06 I^2^ = 0% *p* = 0.01) (Figure 4).

### Risk of Bias Analysis

Our judgment on the risk of bias in the included studies is presented in Table 4. The included RCTs were of moderate-high quality with low risk of bias in majority domains. The non-RCTs had a high risk of bias in the domains of confounding factors and blinding of outcome assessment.

**Table 4:** Risk of bias in included studies.

**RCTs**						
**References**	**Random sequence generation**	**Allocation concealment**	**Blinding of participants and personnel**	**Blinding of outcome assessment**	**Incomplete outcome data**	**Selective reporting**
Nedeljkovic et al. (30)	Low risk	Low risk	Low risk	Low risk	Low risk	Low risk
Wong et al. (31)	Low risk	Unclear risk	Low risk	Low risk	Low risk	Low risk
Ha et al. (32)	Low risk	Low risk	Low risk	Low risk	Low risk	Unclear risk
Hutchins et al. (33)	Low risk	Low risk	Low risk	Low risk	Low risk	Low risk
Hutchins et al. (34)	Low risk	Low risk	Low risk	Low risk	Low risk	High risk
**RETROSPECTIVE STUDIES**						
**References**	**Selection of participants**	**Confounding variables**	**Intervention measurements**	**Blinding of outcome assessment**	**Incomplete outcome data**	**Selective outcome reporting**
Guerra et al. (35)	Low risk	High risk	Low risk	High risk	Low risk	Unclear risk
Stokes et al. (36)	Low risk	High risk	Low risk	High risk	Low risk	Unclear risk

*RCT, randomized control trial*.

## Discussion

RB has one of the longest half-lives among commonly used local anesthetics in clinical practice. However, even with a terminal half-life of 3.5 h pain control by RB may not extend beyond the first postoperative day (12). In this context, LB was developed to prolong the analgesic effect of an already long-acting anesthetic agent. LB is based on the DepoFoam technology, which encapsulates the drug in a liposomal platform and releases them over a period of 1–30 days. In LB, the active drug lies in microscopic spherical liposomes, which are composed of biodegradable cholesterol, triglycerides, and phospholipids. Gradually, these microscopic vesicles reorganize and break open thereby releasing the drug at the injected site over a longer duration. The liposomes do not alter the chemical composition of the drug and also reduce the systemic toxicity by avoiding high drug plasma levels (12, 37). In 2011, the USA Food and drug administration (FDA) first approved the use of LB in wound infiltration for hemorrhoidectomies and bunionectomies. Since then, LB has also been approved for local infiltration in total knee arthroplasty, mammoplasty, inguinal hernia repair, and also as a field block agent in TAP blocks (37). However, whether the pharmacological properties of LB translate into clinically relevant prolonged analgesia with the TAP block is not known and was the subject of this study.

The effect of TAP blocks with regular anesthetic agents is known to last anywhere between 6 and 24 h (34). Ma et al. (38), in their systematic review and meta-analysis of 56 RCTs, have demonstrated that postoperative pain in the first 24 h is significantly reduced by the TAP block administered for various surgical procedures. They also reported that the TAP block significantly reduced morphine consumption and increased the time for the first analgesic request. While several RCTs have assessed the efficacy of regular anesthetics for the TAP block, only limited studies have compared single infiltration of TAP blocks with LB and regular anesthetics for various abdominopelvic surgical procedures. For the first primary outcome of our study i.e., total analgesic consumption, a meta-analysis could not be performed owing to significant differences in data collection and presentation among the included studies. Examining the results by specific periods, out of three studies (30, 31, 36) evaluating analgesic consumption for 0–48 h, only Nedeljkovic et al. (30) reported statistically significant results. Hutchins et al. (33, 34) in their two trials reported analgesic consumption for 24–48 and 48–72 h but with opposite results. For 0–72 h, data were reported only by Nedeljkovic et al. (30) and Hutchins et al. (34) with both reporting reduced analgesic consumption in the LB group. On the other hand, Ha et al. (32) and Guerra et al. (35) reported analgesic consumption for the entire duration of hospitalization but with contrasting results.

The overall incoherent results among the studies of our review can be attributed to many factors. First, TAP blocks were a part of multimodal analgesia plans in the included studies and not singularly used for pain control. The use of additional pain control measures like paravertebral blocks in the enhanced recovery protocol of Ha et al. (32) could have contributed to nonsignificant results. Second, accurate placement of the block is essential for optimal pain relief (30). In the trial of Nedeljkovic et al. (30), around 6% of patients received incorrect TAP blocks and were excluded from the analysis. Since none of the other studies assessed the accuracy of the TAP blocks, its exact influence on the review outcomes cannot be determined. Third, there was interstudy heterogeneity in the technique of TAP (ultrasound vs. laparoscopic) and the dosage of LB and RB. Despite the different techniques, RCTs have demonstrated that laparoscopic and ultrasound-guided TAP blocks have similar efficacy (39, 40). The precise role of variable dosage of RB in the control group and different combinations of LB and RB in the study group is also difficult to ascertain. As for RB, Ng et al. (41) in a meta-analysis of 14 studies were unable to delineate any difference in analgesic efficacy with high dose (>50 mg) or low dose (≤50 mg) RB for TAP blocks when used for cesarean sections. However, no study has measured the relationship of different doses of LB with the duration of pain relief after nerve or field blocks.

For the second primary outcome of our review, our analysis indicated a statistically significant reduction in pain scores with the use of LB at 12, 24, and 48 h. However, the pooled analysis failed to demonstrate any significant differences at 72 h. Further, the effect size for the results was not large with the upper end of 95% CI very close to zero (−0.06 for 24 h and −0.04 for 48 h). The concept of minimum clinically important difference (MCID) for patient-reported measures describes "the smallest difference in score in the domain of interest which participants perceive as beneficial” (42). While the MCID for pain scores after abdominopelvic surgery is not known (43), considering the very small effect size, its clinical relevance may not be high. The results of our study concur with other meta-analysis studies assessing the role of LB. Kolade et al. (14), in a review of LB for shoulder surgeries, have failed to elucidate any significant benefit of LB vs. regular anesthetics for reducing total opioid consumption or pain scores up to 48 h after the procedure. Hamilton et al. (44), in a Cochrane review of 2016, concluded that there is a lack of sufficient data to support or refute the use of LB for peripheral nerve blocks for the management of postoperative pain. Yayac et al. (45), in a recent review of 42 studies on total knee arthroplasty, have reported that despite significantly lower pain scores with LB, better pain control may not be clinically significant, and the use of LB may not reduce total opioid consumption.

One important variable that may limit a more liberal use of LB is its high cost. While some studies report that the better and prolonged pain control offered by LB reduces hospital stay and in turn compensates for the cost of the drug (33, 35), the same has not been duplicated by other clinicians (46, 47). Beachler et al. (47) have indicated that given the minimal clinical benefits of LB and the substantial cost of the drug, its use is not justifiable in total hip arthroplasty patients. In our analysis, despite reduced time to ambulation, the length of hospital stay was similar in both groups. Owing to the lack of monetary data, we cannot comment on the cost effectiveness of the drug for TAP blocks.

The results of our study should be interpreted with the following limitations. Foremost, only a limited number of studies were available for analysis of which only two were non-RCTs. The incorporation of nonrandomized studies may have skewed the results of our review. Second, as discussed earlier, there was significant methodological heterogeneity in the included studies. Further, the outcomes of interest were nonconsistently reported by all included studies. There were variations in time intervals, data presentation (least-square means, medians, or means) with some presenting data only pictorially. Data conversion and graphical extraction of data were undertaken to ensure maximum studies are included in the review. However, this may have introduced bias in our results. This may limit the generalization of our review results in wider clinical settings.

To conclude, current evidence on the role of LB vs. RB for providing prolonged analgesia with TAP blocks is unclear. There is conflicting evidence on the role of LB in reducing total analgesic consumption beyond 24 h. LB may result in a small reduction in pain scores up to 48 h but not at 72 h. Further, high-quality homogenous RCTs are needed to establish high-quality evidence.

## Data Availability Statement

The original contributions presented in the study are included in the article/Supplementary Material, further inquiries can be directed to the corresponding author/s.

## Author Contributions

YZ conceived and designed the study. TX, ZC, ZD, and JW were involved in the literature search and data collection. YZ and SQ analyzed the data. YZ wrote the paper. SQ reviewed and edited the manuscript. All authors read and approved the final manuscript.

## Conflict of Interest

The authors declare that the research was conducted in the absence of any commercial or financial relationships that could be construed as a potential conflict of interest.
