# Supplementary material for: Transversus Abdominis Plane Block With Liposomal Bupivacaine vs. Regular Anesthetics for Pain Control After Surgery: A Systematic Review and Meta-Analysis
Source: Front Surg. 2020 Nov 5;7:596653. doi: 10.3389/fsurg.2020.596653 (PMC7674642; doi:10.3389/fsurg.2020.596653)
Supplement: Supplementary file 1 [file Table_1.DOCX]

Supplementary Table S1: Search strategy and results of PubMed database

| **Search number** | **Query** | **Search Details** | **Results** |
| --- | --- | --- | --- |
| 1 | (Transverse abdominal block) AND (Bupivacaine) | ((((("abdominal muscles"[MeSH Terms] OR ("abdominal"[All Fields] AND "muscles"[All Fields])) OR "abdominal muscles"[All Fields]) OR ("transverse"[All Fields] AND "abdominal"[All Fields])) OR "transverse abdominal"[All Fields]) AND (((("block"[All Fields] OR "blocked"[All Fields]) OR "blocking"[All Fields]) OR "blockings"[All Fields]) OR "blocks"[All Fields])) AND ((("bupivacain"[All Fields] OR "bupivacaine"[MeSH Terms]) OR "bupivacaine"[All Fields]) OR "bupivacaine s"[All Fields]) | 184 |
| 2 | (Transverse abdominal plane) AND (bupivacaine) | ((((("abdominal muscles"[MeSH Terms] OR ("abdominal"[All Fields] AND "muscles"[All Fields])) OR "abdominal muscles"[All Fields]) OR ("transverse"[All Fields] AND "abdominal"[All Fields])) OR "transverse abdominal"[All Fields]) AND ((("aircraft"[MeSH Terms] OR "aircraft"[All Fields]) OR "plane"[All Fields]) OR "planes"[All Fields])) AND ((("bupivacain"[All Fields] OR "bupivacaine"[MeSH Terms]) OR "bupivacaine"[All Fields]) OR "bupivacaine s"[All Fields]) | 141 |
| 3 | (transverse abdominal plane) AND (analgesia) | ((((("abdominal muscles"[MeSH Terms] OR ("abdominal"[All Fields] AND "muscles"[All Fields])) OR "abdominal muscles"[All Fields]) OR ("transverse"[All Fields] AND "abdominal"[All Fields])) OR "transverse abdominal"[All Fields]) AND ((("aircraft"[MeSH Terms] OR "aircraft"[All Fields]) OR "plane"[All Fields]) OR "planes"[All Fields])) AND (("analgesia"[MeSH Terms] OR "analgesia"[All Fields]) OR "analgesias"[All Fields]) | 352 |
| 4 | (transverse abdominal plane) AND (anesthesia) | ((((("abdominal muscles"[MeSH Terms] OR ("abdominal"[All Fields] AND "muscles"[All Fields])) OR "abdominal muscles"[All Fields]) OR ("transverse"[All Fields] AND "abdominal"[All Fields])) OR "transverse abdominal"[All Fields]) AND ((("aircraft"[MeSH Terms] OR "aircraft"[All Fields]) OR "plane"[All Fields]) OR "planes"[All Fields])) AND (((("anaesthesia"[All Fields] OR "anesthesia"[MeSH Terms]) OR "anesthesia"[All Fields]) OR "anaesthesias"[All Fields]) OR "anesthesias"[All Fields]) | 525 |
| 5 | (liposomal bupivacaine) AND (block) | ((((((((((("liposomalization"[All Fields] OR "liposomalized"[All Fields]) OR "liposomally"[All Fields]) OR "liposome s"[All Fields]) OR "liposomes"[Pharmacological Action]) OR "liposomes"[MeSH Terms]) OR "liposomes"[All Fields]) OR "liposomal"[All Fields]) OR "liposome"[All Fields]) OR "liposomic"[All Fields]) OR "liposomized"[All Fields]) AND ((("bupivacain"[All Fields] OR "bupivacaine"[MeSH Terms]) OR "bupivacaine"[All Fields]) OR "bupivacaine s"[All Fields])) AND (((("block"[All Fields] OR "blocked"[All Fields]) OR "blocking"[All Fields]) OR "blockings"[All Fields]) OR "blocks"[All Fields]) | 257 |
